# Hyperprolactinemia in women: treatment

**DOI:** 10.61622/rbgo/2024FPS05

**Published:** 2024-04-25

**Authors:** Cristina Laguna Benetti-Pinto, Andrea Prestes Nácul, Ana Carolina Japur Rosa e Silva, Gustavo Arantes Rosa Maciel, Vania dos Santos Nunes Nogueira, Paula Condé Lamparelli Elias, Manoel Martins, Leandro Kasuki, Heraldo Mendes Garmes, Andrea Glezer

**Affiliations:** 1 Universidade Estadual de Campinas Faculdade de Ciências Médicas Departamento de Obstetrícia e Ginecologia Campinas SP Brazil Departamento de Obstetrícia e Ginecologia, Faculdade de Ciências Médicas, Universidade Estadual de Campinas, Unicamp, Campinas, SP, Brazil; 2 Unidade de Reprodução Humana Hospital Fêmina Grupo Hospitalar Conceição Porto Alegre RS Brazil Unidade de Reprodução Humana, Hospital Fêmina, Grupo Hospitalar Conceição, Porto Alegre, RS, Brazil; 3 Universidade de São Paulo Faculdade de Medicina de Ribeirão Preto Departamento de Ginecologia e Obstetrícia Ribeirão Preto SP Brazil Departamento de Ginecologia e Obstetrícia, Faculdade de Medicina de Ribeirão Preto, Universidade de São Paulo, Ribeirão Preto, SP, Brazil; 4 Universidade de São Paulo Faculdade de Medicina Hospital das Clínicas HCFMUSP Sao Paulo SP Brazil Departamento de Obstetrícia e Ginecologia, Disciplina de Ginecologia, Hospital das Clínicas HCFMUSP, Faculdade de Medicina, Universidade de São Paulo, Sao Paulo, SP, Brazil; 5 Universidade Estadual Paulista Faculdade de Medicina de Botucatu Departamento de Clínica Médica Botucatu SP Brazil Departamento de Clínica Médica, Faculdade de Medicina de Botucatu, Universidade Estadual Paulista (UNESP), Botucatu, SP, Brazil; 6 Universidade de São Paulo Faculdade de Medicina de Ribeirão Preto Hospital das Clínicas São Paulo SP Brazil Hospital das Clínicas, Faculdade de Medicina de Ribeirão Preto, Universidade de São Paulo, São Paulo, SP, Brazil; 7 Universidade Federal do Ceará Núcleo de Pesquisa e Desenvolvimento de Medicamentos Departamento de Medicina Clínica Fortaleza CE Brazil Departamento de Medicina Clínica e Núcleo de Pesquisa e Desenvolvimento de Medicamentos, Universidade Federal do Ceará, Fortaleza, CE, Brazil; 8 Universidade Federal do Rio de Janeiro Hospital Universitário Clementino Fraga Filho Rio de Janeiro RJ Brazil Hospital Universitário Clementino Fraga Filho, Universidade Federal do Rio de Janeiro, Rio de Janeiro, RJ, Brazil; 9 Universidade Estadual de Campinas Faculdade de Ciências Médicas Campinas SP Brazil Faculdade de Ciências Médicas, Universidade Estadual de Campinas, Campinas, SP, Brazil; 10 Universidade de São Paulo Hospital das Clínicas Faculdade de Medicina São Paulo SP Brazil Hospital das Clínicas, Faculdade de Medicina, Universidade de São Paulo, São Paulo, SP, Brazil

## Key points

- Hyperprolactinemia (HPRL) is a cause of menstrual irregularity, galactorrhea, hypogonadism and infertility. Recognizing the cause of HPRL is essential to institute appropriate treatment.
- Drug treatment with dopamine agonist (DA) is the first line of therapy for symptomatic HPRL due to prolactinoma or idiopathic HPRL.
- Dopamine agonist treatment is effective in 80-90% of cases.
- Infertility treatment in patients with HPRL is also carried out with DA, aiming to normalize prolactin (PRL) levels.
- The risk of symptomatic growth of prolactinomas during pregnancy is related to the size of the tumor, length of previous treatment and higher PRL levels, and is less than 5% in microprolactinomas.
- Hyperprolactinemia remission can occur after pregnancy and menopause, and in around 30% of cases following drug treatment.
- The use of drugs such as antidepressants and antipsychotics is a frequent cause of HPRL, and has particularities in its management.

## Recommendations

- The initial treatment of patients with HPRL caused by prolactinoma or idiopathic HPRL is with medication and DAs are the option of choice. Cabergoline (CAB) is more effective and better tolerated than bromocriptine (BRC), representing the first therapeutic choice.
- Treatment should begin with lower doses with slow progression for better tolerability. The use of high doses requires attention to potential side effects and psychiatric effects, as well as monitoring for heart valve disease.
- In cases of good control of symptoms, PRL levels and the size of the prolactinoma with DAs, a minimum of two years of treatment is recommended before starting to reduce the dose and withdraw the medication.
- In symptomatic patients, surgical treatment of prolactinomas may be indicated in cases of intolerance or resistance to DAs.
- The symptomatic growth of microadenomas is not frequent during pregnancy. Dopamine agonist can be withdrawan in cases of idiopathic HPRL, microadenomas and intrasellar macroadenomas that showed good tumor control after at least one year of drug treatment before pregnancy.
- Tumors diagnosed during menacme and with good control with DA can generally have treatment discontinued with menopause.
- The status of the prolactinoma must be re-evaluated with sellar imaging after one to two years of treatment with DA, after pregnancy and after menopause. The exception are macroadenomas with compressive risk or inadequate response that must be individually re-evaluated.
- The patient should be referred to specialists in cases of HPRL by stalk disconnection, acromegaly, resistant and aggressive prolactinoma, and in cases of hypopituitarism.
- For pharmacological causes, the patient should be referred to the prescriber physician so they can evaluate whether to suspend or replace the drug. If this is not possible, pituitary imaging should be performed to rule out a tumor and hormone replacement containing estrogen should be considered to reduce risks whenever there is hypogonadism.

## Background

The treatment of HPRL, when necessary, is preferably carried out with DA, generally for a prolonged period of time. There are particular situations in the female body, since PRL secretion is influenced by the menstrual cycle, menopause and pregnancy. The main ovarian hormone – estrogen – interferes with PRL secretion through different mechanisms: regulation of PRL gene expression, downregulation of dopamine receptor expression and stimulation of lactotroph proliferation. Therefore, estrogen is considered a PRL-releasing factor. During pregnancy, when circulating estrogen levels are high, the production of PRL and the growth of prolactinomas may be stimulated. During menopause, with the reduction in estrogen secretion by the ovaries, there is a reduction in the stimulus they exert on PRL secretion and the proliferation of lactotrophs. Furthermore, PRL is the only pituitary hormone regulated primarily through hypothalamic tonic inhibition via dopamine. Therefore, interruption of the arrival of dopamine by compression or inflammation of the pituitary stalk or by various medications can cause HPRL. Then, in women with confirmed HPRL, determining the etiology and the moment of reproductive life can influence the therapeutic decision.

## When to treat hyperprolactinemia?

Treatment of HPRL with a DA is indicated to control symptoms and the effects of tumor mass in the case of macroprolactinomas.(1)

Treatment indications:

- Symptoms secondary to hypothalamic-pituitary-gonadal axis dysfunction: menstrual irregularity or amenorrhea, infertility, decreased libido
- Bothersome galactorrhea
- Desire for conception
- Neurological symptoms
- Control of tumor mass (macroprolactinomas)

## When not to treat hyperprolactinemia?

The management of microprolactinomas in asymptomatic patients can be discussed and monitored without specific treatment.^(1)^ In patients with amenorrhea secondary to a microprolactinoma who do not wish to become pregnant, combined hormonal contraceptives may be an option to DA.^(1,2)^ In the case of the option for follow-up or use of oral combined hormonal contraceptives, the patient must be guided and involved in the debate regarding the importance of regular monitoring of the tumor image and the possibility of introducing DA if there is tumor growth. In cases of HPRL secondary to hypothyroidism, the thyroid dysfunction must be treated previously and after normalization, serum PRL levels and symptoms should be re-evaluated. Hyperprolactinemia secondary to medications will be covered in another topic.

## How and for how long should hyperprolactinemia be treated?

Dopamine agonists are considered first-line treatment for prolactinomas and idiopathic HPRL.^(1,2)^ They act by binding to the type 2 dopamine receptor (DR2), which is highly expressed in prolactinomas.^(3)^ Two drugs of this class are available in Brazil: bromocriptine (BRC) and cabergoline (CAB). These DAs lead to normalization of PRL levels and reduction in prolactinoma size in most cases. Cabergoline is considered the drug of choice due to its greater efficacy (normalization of PRL levels in 95% vs. 80% of cases with BRC) and better dosage and tolerability. Table 1 brings together the main characteristics of these two medications.

**Table 1:** Main characteristics of the two dopaminergic agonists commercially available in Brazil

	Cabergoline	Bromocriptine
Recommended average doses	0.5 - 2 mg	2.5 – 7.5 mg
Biochemical efficacy	Up to 95%	Up to 80%
Half-life (hours)	63-69	6-20
Usual dosage	Once to twice a week	Two to three times a day

Treatment should be started with 0.25 to 0.5 mg of CAB once a week in microprolactinomas and idiopathic HPRL, and 0.5 mg twice a week in most macroprolactinomas. Levels of PRL should be measured every four to eight weeks of treatment, with progressive dose increase if PRL normalization is not achieved.^(1,2)^ In case of normalization of PRL, sellar magnetic resonance imaging (MRI) can be repeated after one to two years of treatment for microprolactinomas. Re-evaluation using sellar MRI after six months of treatment is appropriate for most macroprolactinomas without compressive symptoms and with a good response to DA treatment, but in case of visual loss, it should be repeated one to three months after starting treatment.^(1,2)^ Remission of prolactinomas with DA treatment is possible and can be achieved mainly after two years of treatment.^(4)^ In a recent meta-analysis, 36.6% of patients maintained remission after discontinuing DAs, reaching 41.2% in patients using CAB.^(5)^ Progressive dose reduction and withdrawal of DA may be indicated after two years of treatment in asymptomatic women with normal PRL levels and absence of residual tumor or important tumor reduction on MRI, with no signs of invasion into the cavernous sinus and a safe distance from the optic pathways. In the first year after suspension, it is recommended to dose PRL every three months, as the risk of relapse is greater, and at longer intervals after this period.^(1,2)^ If HPRL recurs, a second attempt to withdraw the drug may be recommended after two more years of treatment and with normal PRL levels.^(6)^

## What are the challenges related to drug treatment?

The main challenges related to the drug treatment of HPRL are intolerance and resistance to DAs.

## What are the mechanisms and frequency of intolerance to DAs and the most common adverse events?

The most common adverse events of DAs are related to their action on dopamine receptors, namely dizziness, postural hypotension, nausea, vomiting, headache, constipation and gastroesophageal reflux (Table 2). ^(7)^ Most of them are dose dependent.

**Table 2:** Adverse events of dopaminergic agonists

Most common events	Nausea
	Headache
	Dizziness, postural hypotension
	Abdominal pain, dyspepsia
	Fatigue
	Nasal congestion
Occasional events	Vomiting
	Constipation
	Leg cramps
Rare Events	Insomnia
	Raynaud's phenomenon
	Heat waves
	Thromboembolic phenomena*
	Heart valve disease
	Depression, anxiety, psychosis
	Pleural/pulmonary fibrosis
	Constrictive pericarditis
	Dyskinesia
	Paresthesia
	Psychiatric events: compulsive
	behaviors

* No causal relationship has been demonstrated to date.

Source: Adapted from Olafsdottir and Schlechte (2006).^(8)^

The safety profile of CAB is similar to that reported for BRC, but adverse events are generally less frequent (Table 3), less serious and of shorter duration and disappear with dose reduction or continued use in many patients, which reflects in discontinuation rates of 12% in BRC treatments vs. 3% in CAB use.^(9,10)^

**Table 3:** Adverse events in women with HPRL treated with BRC or CAB

		% in BRC n = 251	% in CAB n = 221
Gastrointestinal tract	Nausea	50	31
	Vomiting	10	4
	Constipation	9	7
	Dry mouth, dyspepsia, reflux	Rare	
Cardiovascular system	Postural hypotension	26	25
	Flushing, nasal congestion	Rare	
Neurological system	Headache	29	30
	Psychosis, mania, paresthesia, nightmare, blurred vision	Rare, high doses	

HPRL - hyperprolactinemia; CAB - cabergoline; BRC - bromocriptine

Source: dos Santos Nunes et al. (2011)^(9)^ and Webster et al. (1994).^(10)^

## How can we prevent or minimize the most common adverse events?

It is recommended to begin treatment with lower doses, after a meal, with gradual increase.^(11)^ Serotonin receptor antagonist antiemetics (for example, ondansetron) should not be taken together, as they may increase the hypotensive effect of dopamine agonists.

## Heart valve disease

The cardiac safety of DAs was questioned after the establishment of an association with valvulopathy in patients with Parkinson's disease.^(12)^ The pathophysiology is due to the proliferation of fibroblasts under the valves and tendon chordae by an agonist action on the 5HT2B serotonin receptor. Cabergoline is an agonist of this receptor and BRC is a partial agonist. Patients with Parkinson's disease are, in general, older in age, have more comorbidities and receive much higher doses of CAB than those used in the treatment of HPRL. Currently, the CAB leaflets in Brazil suggest an echocardiographic evaluation before starting to use the medication and in some countries, such as the United States, there is a recommendation for periodic echocardiographic evaluation. Several studies have been published since 2008. Most show that commonly used doses (CAB up to 2.0 mg/week) were not associated with clinically significant valvular heart disease, although a 2018 meta-analysis including 13 studies with CAB for HPRL used for at least six months indicated increased risk of mild or clinically significant (moderate or severe) tricuspid insufficiency.^(13)^

## Therefore, considering the cost-benefit, who should perform an echocardiogram?

When starting treatment for HPRL with DA, a clinical assessment of cardiac risk is indicated through medical history and physical examination and, at clinical discretion, echocardiographic evaluation in patients at higher risk, if available. In patients with moderate/severe valve abnormalities with clinical repercussions, CAB should be avoided. For most patients who have good control and tolerate DAs at standard doses, the risk of developing valvular heart disease appears to be very low. In patients with resistant prolactinomas, who require higher doses (>2.0 mg/week), we suggest vigilance, considering periodic echocardiography.^(1,2,14)^

## Mood changes and impulse control disorders

Patients with HPRL appear to have reduced quality of life and different personality profiles compared to control individuals, in addition to increased anxiety and depression. There are reports of association between DA treatment with severe depression, manic episodes, and even psychosis. Impulse control disorders are characterized by impulsive behaviors that interfere with the individual's daily life and can be characterized by compulsive buying, compulsive eating, pathological gambling, punding (performance of repetitive tasks), hypersexuality, among others. The risks and benefits must be assessed together with a psychiatrist to decide on the best course of action: maintain DA at lower doses and psychiatric monitoring/treatment, or indication of neurosurgery in cases with tumor growth.^(15,16)^

## Rare complications of clinical treatment

Cerebrospinal fluid fistula is a rare event in prolactinomas, generally associated with macroprolactinomas with invasion of the sphenoid sinus and related to the beginning of DA treatment (weeks to months). It can occur spontaneously or after tumor reduction. It is believed that the tumor works like a cork and once necrosis of the tumor tissue occurs, the lesion will no longer be able to block the flow of fluid, and leakage may occur. Treatment to correct the fistula must be surgical in order to avoid meningitis.^(17)^

Apoplexy is characterized by acute infarction and/or hemorrhage of the pituitary gland, leading to an abrupt increase in tissue volume in the sellar region, which can cause headache, visual impairment, cranial nerve palsy and even impaired consciousness, in addition to causing pituitary hormone deficiencies. Among pituitary tumors, prolactinoma is the most commonly related to apoplexy, and the use of DA and pregnancy are risk factors. Although it is a rare event, new-onset headache or sudden worsening during prolactinoma treatment indicates urgent sellar imaging for the correct diagnosis. Management must be carried out by a multidisciplinary team.^(18)^

Rarely, there may be worsening of vision during DA treatment, despite normalization of PRL levels. One of the causes is herniation of the optic chiasm, which can be diagnosed by MRI of the sellar region. Withdrawal or dose reduction of DA and neurosurgery with chiasmopexy may be indicated after individual assessment by a multidisciplinary team.^(17)^

## What are the contraindications to the use of dopaminergic agonists?

Contraindications to DAs include: breastfeeding, hypersensitivity to ergotamine derivatives, uncontrolled high blood pressure, severe cardiovascular disease, history of untreated coronary disease and uncontrolled psychiatric disease.

## How to define resistance to dopaminergic agonists?

There is still no universal consensus on the definition of DA resistance and dose. Regarding hormonal response, various criteria have been used, such as failure to normalize PRL levels, failure to reduce PRL levels enough to achieve ovulation, or failure to allow a 50% reduction in HPRL.^(1)^ However, as the threshold of PRL reduction required to allow normal function of the gonadal axis varies with each individual, it seems sensible to define hormonal resistance to DAs as the failure to achieve normoprolactinemia or symptom control. For macroprolactinoma, resistance to DAs may be considered when there is little or no impact on tumor size. Various criteria have been used, including less than 30% reduction in maximum tumor diameter.

Resistance to DAs occurs in 20-30% of prolactinomas. Of note, an additional responses to DAs may occur in the long term. Not all women with resistance to DAs require a change in treatment, such as asymptomatic women without mass effect with stable adenoma.

## What are the mechanisms and clinical factors associated with resistance to DAs?

The main mechanism of resistance occurs through reduced expression of the dopamine receptor subtype 2 (D2R), particularly its short isoform. Younger age at diagnosis (children and adolescents), male sex, tumors with invasion into cavernous sinuses and predominantly cystic tumors are the factors most related to resistance to DAs.^(19)^ In the presence of adequate adherence to clinical treatment and loss of response (secondary resistance), referral to a reference center is necessary to investigate changes in biological behavior and malignant transformation.

## What is the management in cases of resistance?

**Figure 1:**
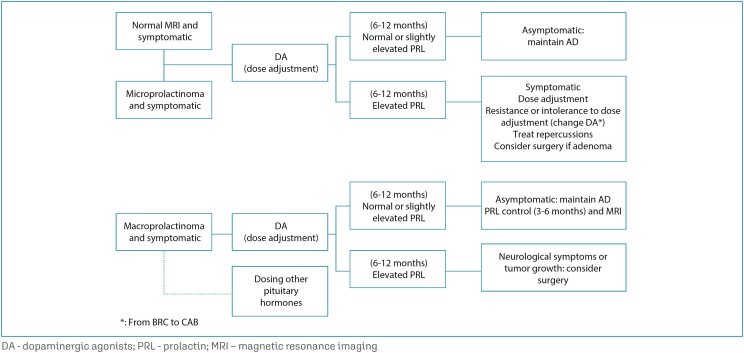
Flowchart of treatment of idiopathic hyperprolactinemia and prolactinomas

If the patient is using BRC, switching to CAB is recommended.^(1,20)^ Cases of resistance to CAB that respond to BRC are occasional.^(1,20)^ In cases of partial resistance with doses of up to 2 mg/week of CAB and tolerance to the drug, larger doses can be used. Patients should be informed about the potential long-term side effects of high doses, in particular the risk of cardiac valve fibrosis. It is also advisable to try reducing the dose after achieving PRL normalization. Little benefit was shown with doses greater than 3.5 mg/week, which is the "maximum" value to be used under surveillance for side effects.^(19)^ Totally resistant or partially resistant patients can benefit from neurosurgical treatment of prolactinoma usually via the transsphenoidal route. The surgery must be performed in a reference center for the treatment of pituitary tumors with an experienced multidisciplinary team (Figure 1).^(21)^

## How to manage hyperprolactinemia in infertility and pregnancy?

**Figure 2:**
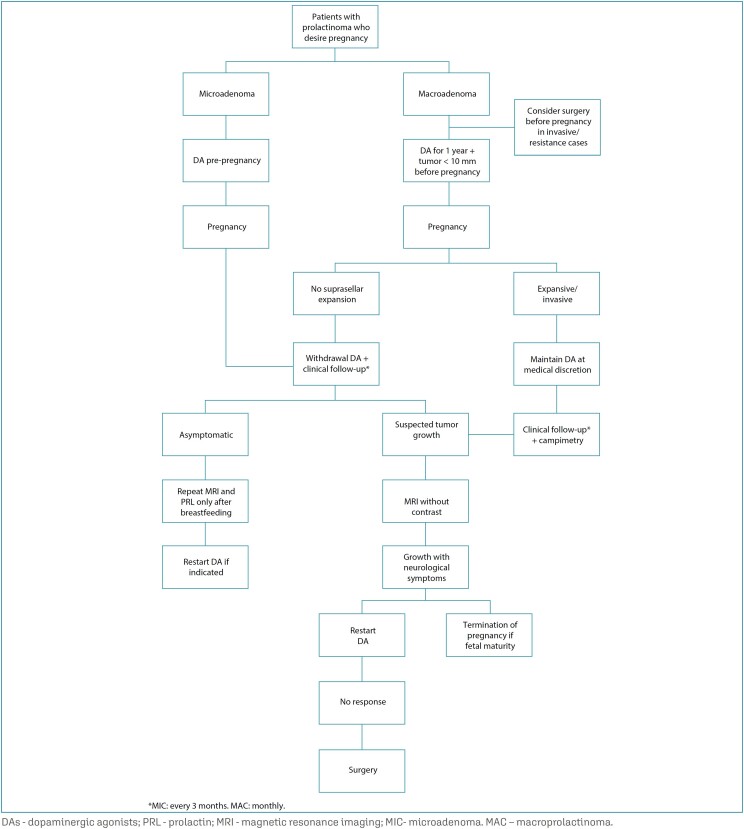
Flowchart of management of prolactinomas during pregnancy

Hyperprolactinemia is present in 15-20% of infertile women. It is characterized by anovulatory cycles, oligomenorrhea and/or amenorrhea, and luteal phase insufficiency.^(22)^ Although BRC is more studied in pregnant women, CAB appears to be more effective in restoring fertility, with better tolerability. To date, there are more than 950 pregnancies induced with CAB described in the literature, showing no increase in the frequency of spontaneous abortion, premature birth, multiple pregnancies or neonatal malformations.^(23)^ Physical or developmental abnormalities have not been described either in follow-up studies of approximately 230 children followed up until 12 years of age after fetal exposure to CAB.^(24)^ In cases where there is no return of ovulation and persistence of high PRL levels in women with prolactinoma, despite the maximum tolerable dose of CAB , the possibility of transsphenoidal surgery can be discussed with the patient. In this case, the patient must be informed about the potential risk of hypopituitarism after surgery with its consequences for fertility.^(25)^ When both treatments do not restore ovulation, ovulation induction with clomiphene citrate or gonadotropins can be considered, ideally after reaching normal PRL levels.^(26)^ Hyperplasia of normal lactotrophs and tumor cells may occur during pregnancy as a result of hyperestrogenism.^(24,27)^ Despite this effect of pregnancy on prolactinomas, the real chance of microadenoma growth, leading to symptoms such as headache and/or visual changes is around 2.5% of cases, whereas in macroprolactinomas not previously operated or irradiated, it can reach almost 20%, which is why management differs according to tumor size.^(24)^ Furthermore, macroprolactinomas have a considerable clinical risk of apoplexy during pregnancy.^(25)^ The duration of treatment with DA prior to pregnancy (>1 year), small tumors and lower PRL values pre-pregnancy reduce the risk of growth and increase the chance of tumor regression after pregnancy and lactation. In line with these data, in a recent Brazilian multicenter study evaluating 233 pregnancies induced with the use of CAB, 194 women with prolactinoma demonstrated that a shorter period of treatment before pregnancy (<2 years) was associated with significantly higher rates of tumor growth.^(23,28)^ Therefore, considering the low risk of microadenoma growth during pregnancy, the recommendation is to suspend treatment after pregnancy is confirmed.^(1,27)^ In patients with macroadenomas who wish to become pregnant, it is recommended to use DAs for at least one year with the aim to reduce tumor dimensions below 10 mm. If there is tumor reduction, it is possible to discuss discontinuing the medication during pregnancy. In cases with suprasellar expansion or that do not respond to DA treatment, transsphenoidal surgery before pregnancy should be considered.^(24)^ Pregnant women with operated macroadenomas have a chance of tumor expansion or growth equal to that of women with microadenomas, i.e., around 2.5% of cases. In cases of women who became pregnant while using DA therapy for a short period without reducing the size of the tumor or who had not previously undergone surgery or radiotherapy, the maintenance of medication should be considered and discussed with the patient, especially if the tumor is close to or in contact with the optic pathways.^(1)^ In these cases, the medication must be maintained at the same dose used pre-pregnancy (Figure 2).

Both BRC and CAB cross the placental barrier. In a European study, a significant increase in pregnancy loss and prematurity was described in women exposed to both BRC and CAB, compared to unexposed women, suggesting an effect related to the class of medications and not a specific effect of CAB.^(29)^ In a study published in 2020,^(23)^ the miscarriage rate in women who discontinued CAB shortly after pregnancy diagnosis was lower (7.5%) than in those who maintained the medication, either on medical advice or inadvertently (38%). The woman's age at pregnancy, PRL levels, CAB dose, gestational age at CAB discontinuation or the presence of comorbidities were not associated with abortion rates. Despite this potential effect on abortion rates, there was no association between maintaining CAB beyond the first trimester and preterm birth, congenital malformations, or neurodevelopmental changes.^(23)^

In cases where medication has been suspended, there is no need for differentiated antenatal care. Monitoring signs of tumor growth can be done through anamnesis directed at changes in the visual field or the appearance of frequent headaches, preferably every three months in microadenomas and monthly in macroadenomas. Eventually a neurological evaluation may be performed. In cases of macroadenomas and decision to discontinue the medication, quarterly visual perimetry should be considered, regardless of the appearance of neurological symptoms. Prolactin dosing during pregnancy is not indicated, since an increase of up to 10 times in basal PRL levels is physiological. The routine request for a serial central nervous system imaging examination should not be done either. This indication is restricted to situations in which there is suspicion of tumor expansion, suggested in the presence of changes in the visual field reported by the patient or visual campimetry, or the onset of frequent headaches. Given the need to perform MRI of the sella turcica, contrast, such as gadolinium, should not be used. Even in cases where, after stopping the medication, symptoms return and tumor growth is confirmed, the medication must be reintroduced.^(1)^ In the absence of response to drug treatment, decompression surgery may be indicated, preferably in the second trimester or evaluate the termination of pregnancy at a more advanced gestational age.^(24,25)^

In healthy women, PRL levels are normalized one week after birth if there is no breastfeeding.^(30)^ Although nipple sucking stimulates PRL pulses, serum values during this period are not usually elevated. Therefore, breastfeeding is not contraindicated in women with prolactinoma, except in those with signs of tumor expansion.^(24)^ When inhibition of lactation is necessary, CAB 1.0 mg (2 tablets) in a single dose can be prescribed in the immediate postpartum period or, in cases of established lactation, use 0.25 mg (half a tablet) every 12 hours for two days, in a total dose of 1 mg, with lactation suppression of 78% to 94%.^(31)^

## Hyperprolactinemia and menopause: any changes in monitoring?

Estrogen is a PRL-releasing factor. With the reduction in estrogen production during menopause, there is a reduction in the stimulus it exerts on PRL secretion and the proliferation of lactotrophs, in addition to changes in warning symptoms for the diagnosis of HPRL resulting from the interruption of menstrual cycles. Although all these changes are expected, there is a lack of evidence on the relationship between the hormonal changes during this period and the production of PRL.^(32-34)^

## Does menopause change the management in women with hyperprolactinemia diagnosed during the reproductive period?

Considering the state of physiological hypoestrogenism, asymptomatic postmenopausal women diagnosed with microadenoma or idiopathic HPRL during reproductive life can do without DA treatment. On the other hand, those with macroprolactinomas, depending on the size of the tumor and behavior during treatment, with less volume reduction, may require continued medication to avoid compressive effects.^(33,35)^

In a study that followed 11 women with microadenoma, there was remission of HPRL in 45% after menopause.^(36)^ In another multicenter study, 29 postmenopausal women diagnosed with prolactinoma during menacme (22 with microadenomas and seven with macroadenomas) were included. In microadenomas, DA was suspended in 90% of cases and there was remission of HPRL and reduction of the tumor, and in 50% there was no residual tumor. In macroadenomas in which treatment was suspended, PRL levels showed a slight increase above the normal range.^(37)^ In a third study, 28 women with micro and macroadenomas who had DA treatment interrupted in post-menopause were evaluated. The PRL levels increased, reduced but did not normalize or remained normal, respectively, in 15%, 33% and 52% of cases. There was no relationship between remission and duration of previous treatment, PRL level or tumor size at the time of diagnosis or discontinuation of DA. The authors also reported tumor growth in 7% of cases.^(38)^

Thus, in women diagnosed during the reproductive period with good HPRL control and tumors without evidence of compressive effect, DA treatment can be suspended after menopause. The remission of HPRL in these cases has been documented in up to two thirds of cases and recurrence in approximately 33% of cases. Regarding tumor size, disappearance and stabilization were reported, respectively, in 58% and 59% of women, with growth in less than 3% of cases.^(33,35)^ Regarding macroprolactinomas, in selected cases with large volume reduction and no apparent risk of compression of the optic chiasm, the withdrawal of medication is suggested, maintaining careful control of the tumor.^(35)^

After menopause and discontinuation of DA, we suggest follow-up with clinical evaluation and PRL dosage every six months in the first year and pituitary MRI at 12 months or earlier if there are symptoms of tumor mass effect. In expansive and/or invasive tumors, it is suggested to maintain the use of DA.

## What changes in relation to prolactinomas diagnosed after menopause?

The true incidence of prolactinomas diagnosed only after menopause is unknown. In general, the diagnosis is made by the presence of neurological symptoms, serum PRL levels are generally quite high, macroadenomas are more frequent and, even with late diagnosis, there is a good response to the prescription of DA.^(39)^

## How to manage HPRL secondary to medications?

Of the various medications that cause HPRL, antipsychotics are the most relevant, as they cause HPRL with greater frequency and intensity. Furthermore, these medications must be managed by a psychiatrist, and a general practitioner or other specialty physician must not suspend them without prior contact with the prescriber. The treatment of HPRL is indicated when associated with hypogonadotropic hypogonadism, due to the repercussions of hypoestrogenism. In this situation, three options are possible: (1) change the medication that induces HPRL, (2) undergo hormonal replacement therapy with estrogen associated with progestogen (the latter in the case of a present uterus), (3) prescribe DA, but in this case, close follow-up is necessary due to the risk of exacerbation of the psychotic condition due to the opposing effect between psychiatric medication and DAs.^(40)^ A recent review of the literature showed that patients with antipsychotic-induced HPRL may benefit from replacement or association with aripiprazole, an antipsychotic that does not cause HPRL.^(40)^

## Final considerations

This text was prepared jointly by the Department of Neuroendocrinology of SBEM and the National Specialized Commission on Endocrine Gynecology of FEBRASGO. It aims to update and assist gynecologists, endocrinologists and primary care physicians in the treatment of patients with HPRL.

## National Specialized Commission on Gynecological Endocrinology of the Brazilian Federation of Gynecology and Obstetrics Associations (FEBRASGO)

### President:

Cristina Laguna Benetti Pinto

Vice president:

Ana Carolina Japur de Sa Rosa e Silva

### Secretary:

José Maria Soares Júnior

### Members:

Andrea Prestes Nácul

Daniela Angerame Yela

Fernando Marcos dos Reis

Gabriela Pravatta Rezende

Gustavo Arantes Rosa Maciel

Gustavo Mafaldo Soares

Laura Olinda Rezende Bregieiro Costa

Lia Cruz Vaz da Costa Damásio

Maria Candida Pinheiro Baracat Rezende

Sebastião Freitas de Medeiros

Tecia Maria de Oliveira Maranhão

Vinicius Medina Lopes
